# A Novel Fuzzy Parameterized Fuzzy Hypersoft Set and Riesz Summability Approach Based Decision Support System for Diagnosis of Heart Diseases

**DOI:** 10.3390/diagnostics12071546

**Published:** 2022-06-24

**Authors:** Atiqe Ur Rahman, Muhammad Saeed, Mazin Abed Mohammed, Mustafa Musa Jaber, Begonya Garcia-Zapirain

**Affiliations:** 1 Department of Mathematics, University of Management and Technology, Lahore 54000, Pakistan; aurkhb@gmail.com (A.U.R.); muhammad.saeed@umt.edu.pk (M.S.); 2 College of Computer Science and Information Technology, University of Anbar, Ramadi 31001, Iraq; 3 Department of Computer Science, Dijlah University College, Baghdad 00964, Iraq; mustafa.musa@duc.edu.iq; 4 Department of Medical Instruments Engineering Techniques, Al-Farahidi University, Baghdad 10021, Iraq; 5 eVIDA Laboratory, University of Deusto, Avda/Universidades 24, 48007 Bilbao, Spain

**Keywords:** Riesz Summability, soft set, fuzzy soft set, fuzzy parameterized fuzzy soft set, hypersoft set, decision-making, aggregation operator, Cleveland dataset

## Abstract

Fuzzy parameterized fuzzy hypersoft set (Δ-set) is more flexible and reliable model as it is capable of tackling features such as the assortment of attributes into their relevant subattributes and the determination of vague nature of parameters and their subparametric-valued tuples by employing the concept of fuzzy parameterization and multiargument approximations, respectively. The existing literature on medical diagnosis paid no attention to such features. Riesz Summability (a classical concept of mathematical analysis) is meant to cope with the sequential nature of data. This study aims to integrate these features collectively by using the concepts of fuzzy parameterized fuzzy hypersoft set (Δ-set) and Riesz Summability. After investigating some properties and aggregations of Δ-set, two novel decision-support algorithms are proposed for medical diagnostic decision-making by using the aggregations of Δ-set and Riesz mean technique. These algorithms are then validated using a case study based on real attributes and subattributes of the Cleveland dataset for heart-ailments-based diagnosis. The real values of attributes and subattributes are transformed into fuzzy values by using appropriate transformation criteria. It is proved that both algorithms yield the same and reliable results while considering hypersoft settings. In order to judge flexibility and reliability, the preferential aspects of the proposed study are assessed by its structural comparison with some related pre-developed structures. The proposed approach ensures that reliable results can be obtained by taking a smaller number of evaluating traits and their related subvalues-based tuples for the diagnosis of heart-related ailments.

## 1. Introduction

The customary theory of reasoning is not constantly pertinent in everyday life circumstances, where the handy information is indistinct or rough. To cope with such variety of circumstances, a definite category of sets called fuzzy sets (*f*-sets) (put forward by Zadeh [1]) was observed as suitable. In such sets, each entity of universal set is stated by a belonging grade within [0, 1]. Nevertheless, to handle situations with more complication and hesitation, it was examined that *f*-sets portrayed some inadequacy for the justification with some parameterization modes. To deal with this insufficiency, Molodtsov [2] developed soft sets (*s*-sets) as a new arithmetical parameterized structure. In *s*-sets, every attribute is mapped to the power set of universal set while characterizing approximate function. A novel model of fuzzy soft sets (fs-sets) [3,4] was conceptualized by hybridizing *f*-sets and *s*-sets. Ali et al. [5], Li et al. [6], Maji et al. [7], Pei et al. [8], and Sezgin et al. [9] discussed the rudiments of *s*-sets with numerical examples. Babitha et al. [10,11] introduced the concept of relations, functions, and orders under soft set environment. The researcher [12,13] made rich contributions to the applications of *s*-set hybrids in decision making (DM).

Various real-life states of affairs demand the categorization of attributes into their respective subattributive nonoverlapping sets. The classical literature on *s*-sets is not capable for these situations; therefore, Smarandache [14] introduced hypersoft sets (hs-sets) to deal with insufficiencies of *s*-sets and to handle the environments with multiargument approximate function (maa-function). The basic axiomatic and algebraic properties of hs-sets have been investigated in [15] and explained by numerical examples. Ihsan et al. [16] discussed the validity of hs-sets for the entitlement of multidecisive opinions under expert set environment. Rahman et al. [17,18,19,20,21,22,23] explored the blended operational aspects of hs-sets by considering settings such as complex setting, convexity and concavity setting, parameterization setting, rough setting, and bijection setting. They utilized algorithm-based techniques to resolve real-world DM issues. Saeed et al. [24,25,26,27,28,29] characterized the novel notions of neutrosophic hypersoft mappings, complex multifuzzy hs-set, and neutrosophic hypersoft graphs with applications in decision-making and clinical diagnosis. Saqlain et al. [30,31,32] discussed decision-making techniques for neutrosophic hs-set with the help of aggregation operators and accuracy functions. Recently, Rahman et al. [33,34] made significant additions in the literature of hs-set by using its hybridized models in medical diagnosis and material selection, respectively.

A rapid increase has been reported in heart-related diseases due to substandard edibles, lack of physical exercises, and dull routine of work. The problem of diagnosing heart-related diseases has become crucial and critical. Several deaths have been reported roughly in every part of the world due to such diseases. This has drawn the attention of researchers and cardiologists to carve out various techniques to overcome this problem. Due to the involvement of various factors, it is very hard to identify the exact reasons for such diseases; therefore, most experts usually prefer to assess the susceptibility of patients for such diseases by using various techniques due to their severity. The researchers [35,36,37,38] made significant contributions by introducing various techniques to observe the behavior of electroencephalogram signals that are very helpful for the above mentioned problem.

### 1.1. Research Gap and Motivation

The concept of fuzzy parameterization is in fact intended for allocating the fuzzy grade to each attribute (or subattribute) in the domain of single-argument (or multiargument) approximate function. This concept has been discussed by several researchers [39,40,41,42,43,44,45,46,47,48] using soft-set-like models. In these models, fuzzy parameters are taken as elements in the domain of soft approximate mapping and fuzzy subsets are taken as elements in its codomain. Recently, the researchers [49,50,51,52,53,54] discussed the concept of fuzzy parameterization in matrices under soft set environment. They characterized various new properties and operations with matrix setting and applied them in decision-making, spaces, and numerical data classification. It can easily be observed that these models are unable to tackle the following settings collectively:

1. The hypersoft setting, which demands the categorization of parameters into their relevant subclasses containing their subparametric values; such kind of classification can only be managed by employing maa-function, which takes the Cartesian product (C-product) of subparametric-valued classes as its domain and then approximates them for universal set.
2. Riesz Summability setting, which is capable of tackling the sequential nature of data.

The existing literature is unable to provide any model that may address these limitations collectively. This scarcity of literature is the main source of inspiration for this research. The proposed study is an integrated study of two models: fuzzy parameterized fuzzy hypersoft set (Δ-set) and Riesz Summability. This integration is capable of coping with the above mentioned settings collectively for the diagnosis of heart diseases by taking real data from Cleveland dataset (CD-set).

### 1.2. Significant Contributions

The significant contributions of the study are outlined as follows:

1. An innovative model fuzzy parameterized fuzzy hypersoft set (Δ-set) is characterized and some of its axiomatic cum algebraic properties are investigated. This model employs maa-function with fuzzy parametric tuples as its domain and collection of fuzzy subsets as its codomain;
2. The classical concept of Riesz mean is reviewed and modified for hs-settings;
3. The real attributes of CD-set are analyzed for heart-based ailments analysis and only those of them are opted that have a pertinent role for the adopted model;
4. In order to have their respective attribute values, the operational roles of all opted attributes are discussed along with description on their measuring units;
5. The opted traits and their subvalues are changed to fuzzy values by employing a suitable algebraic technique;
6. Two algorithms (one for aggregations of Δ-set and other for Riesz mean) are proposed and implemented in real-world scenario of medical diagnosis for heart diseases based on fuzzy-valued attributes of CD-set.

## 2. Preliminaries

In this segment of the paper, the necessary definitions are recollected to make the proposed concept clear to readers. The symbols $\ddot{U}$, I, and $P(\ddot{U})$ stand for initial universe, closed unit interval, and power set, respectively, throughout the paper.

**Definition** **1** ([1]). *Af*-set *P is characterized by $P=\{\left(\hat{u},A_{P}\left(\hat{u}\right)\right)|\hat{u}\in \ddot{U}\}$ with $A_{P}:\ddot{U}\to I$ and the value $A_{P}\left(\hat{u}\right)$ is recognized as grade of membership with respect to $\hat{u}\in P$.*

**Definition** **2** ([1]). *Let $P_{1}$ and $P_{2}$ are two f-sets. The f-set $P_{1}$ is said to be subset of other f-set $P_{2}$, denoted by $P_{1}\subseteq P_{2}$, if $A_{P_{1}}\left(\hat{u}\right)\leq A_{P_{2}}\left(\hat{u}\right)$.*

**Definition** **3** ([1]). *The union of two f-sets $P_{1}$ and $P_{2}$ is also a f-set P, denoted by $P_{1}\cup P_{2}$, such that its membership grade $A_{P}$ is given as $A_{P}\left(\hat{u}\right)=Max\left\{A_{P_{1}}\left(\hat{u}\right),A_{P_{2}}\left(\hat{u}\right)\right\}$ for all $\left(\hat{u}\right)\in \ddot{U}$.*

**Definition** **4** ([1]). *The intersection of two f-sets $P_{1}$ and $P_{2}$ is also a f-set P, denoted by $P_{1}\cap P_{2}$, such that its membership grade $A_{P}$ is given as $A_{P}\left(\hat{u}\right)=Min\left\{A_{P_{1}}\left(\hat{u}\right),A_{P_{2}}\left(\hat{u}\right)\right\}$ for all $\left(\hat{u}\right)\in \ddot{U}$.*

**Definition** **5** ([1]). *The complement of a f-set P is also a f-set, denoted by $P^{c}$, such that its membership grade $A_{P^{c}}$ is given as $A_{P^{c}}\left(\hat{u}\right)=1-A_{P}\left(\hat{u}\right)$ for all $\left(\hat{u}\right)\in \ddot{U}$.*

**Definition** **6** ([2]). *If E is a set containing attributes, then the family of pairs $\left(F_{S},G\right)$ is called* *s*-set *on $\ddot{U}$, in which $F_{S}:G\to P\left(\ddot{U}\right)$ and G⊆E.*

**Definition** **7** ([7]). *Union of two s-sets $\left(M_{S_{1}},Z_{1}\right)$ and $\left(M_{S_{2}},Z_{2}\right)$ is a s-set $\left(M_{S_{3}},Z_{3}\right)$ with $Z_{3}=Z_{1}\cup Z_{2}$ and for $\hat{z}\in Z_{3}$,*

$$M_{S_{3}}\left(\hat{z}\right)=\left\{\begin{array}{cc} M_{S_{1}}\left(\hat{z}\right) & \hat{z}\in \left(Z_{1}\setminus Z_{2}\right)\\ M_{S_{2}}\left(\hat{z}\right) & \hat{z}\in \left(Z_{2}\setminus Z_{1}\right)\\ M_{S_{1}}\left(\hat{z}\right)\cup M_{S_{2}}\left(\hat{z}\right) & \hat{z}\in \left(Z_{1}\cap Z_{2}\right) \end{array}\right..$$

**Definition** **8** ([7]). *Intersection of two s-sets $\left(M_{S_{1}},Z_{1}\right)$ and $\left(M_{S_{2}},Z_{2}\right)$ is a s-set $\left(M_{S_{3}},Z_{3}\right)$ with $Z_{3}=Z_{1}\cap Z_{2}$ and for $\omega \in Z_{3}$, $M_{S_{3}}\left(\omega \right)=M_{S_{1}}\left(\omega \right)\cap M_{S_{2}}\left(\omega \right)$.*

Additional description on *S*-set and its operational properties can be reviewed in [3,7].

**Definition** **9** ([14]). *If E is a set containing attributes and H is a collection consisting of the C-product of nonoverlapping subclasses having subattributive values, then the family of pairs (W,H) is known as hs-set on $\ddot{U}$ with $W:H\to P(\ddot{U})$.*

**Definition** **10** ([14]). *A hs-set (W,H) is stated as fuzzy hs-set when $P(\ddot{U})$ in $W:H\to P(\ddot{U})$ is substituted with $F(\ddot{U})$, where $F(\ddot{U})$ is the family of f-sets.*

The references [14,15] are very useful for consulting more operational properties of hs-set.

**Definition** **11** ([39]). *A fpfs-set $R_{S}$ is stated as $R_{S}=\left\{\left(\frac{{ℵ}_{S}\left(\ddot{a}\right)}{\ddot{a}},\hbar_{S}\left(\ddot{a}\right)\right);{ℵ}_{S}\left(\ddot{a}\right)\in F\left(\ddot{U}\right),\hbar_{S}\left(\ddot{a}\right)\in F\left(\ddot{U}\right)\;\;\&\;\;\ddot{a}\in A\right\}$, where A⊆E, ${ℵ}_{S}:A\to I$ and $\hbar_{S}:A\to F\left(\ddot{U}\right)$.*

**Definition** **12** ([55,56]). *If ${\left({\hat{x}}_{p}\right)}_{1}^{n}$ is a sequence with $X_{n}=\sum_{p=1}^{n}{\hat{x}}_{p}$ and $n,{\hat{x}}_{p}\in N^{+}$, then the matrix $M^{\hat{x}}=\left[m_{np}^{\hat{x}}\right]$ of Riesz mean is stated as*

(1) $$m_{np}^{\hat{x}}=\left\{\begin{array}{c} \frac{{\hat{x}}_{p}}{X_{n}}\;,\;\;p\in \left[0,1\right]\\ 0\;\;\;\;\;,\;\;\;\;\;\;\;\;\;p>n \end{array}\right.\;\;\;.$$

*The necessary and sufficient condition for regularity of Riesz mean is $X_{n}\to \infty$ when n→∞. Furthermore, every Riesz mean follows limitation method discussed in [57] and $\sum_{p=1}^{\infty }\left|m_{np}^{\hat{x}}\right|=1.$ For sequence ${\left({\hat{y}}_{p}\right)}_{1}^{n}$, Riesz transform is given as*

(2) $${\hat{z}}_{n}=\sum_{p=1}^{n}\frac{{\hat{x}}_{p}{\hat{y}}_{p}}{X_{n}}\;$$

**Example** **1.** *If we take n=5 in Equation (2), we have*

$${\hat{z}}_{5}=\frac{{\hat{x}}_{1}{\hat{y}}_{1}+{\hat{x}}_{2}{\hat{y}}_{2}+{\hat{x}}_{3}{\hat{y}}_{3}+{\hat{x}}_{4}{\hat{y}}_{4}+{\hat{x}}_{5}{\hat{y}}_{5}}{{\hat{x}}_{1}+{\hat{x}}_{2}+{\hat{x}}_{3}+{\hat{x}}_{4}+{\hat{x}}_{5}}$$

## 3. Fuzzy Parameterized Fuzzy Hypersoft Set (Δ-Set)

The aim of this part is to present the characterization of basic notions of Δ-set introduced by Rahman et al. [48] as a generalization of the concepts stated in [39,43,44] with some modifications. Let $B_{i},i=1,2,\ldots ,n$ be parameter-valued sets for parameters ${\ddot{\partial }}_{i}\in E$ (a set of parameters) with $B_{i}\cap B_{j}=\emptyset ,{\ddot{\partial }}_{i}\neq {\ddot{\partial }}_{j},i\neq j$ and $B=\prod_{i=1}^{n}B_{i}=B_{1}*B_{2}*\ldots *B_{n}$. The notations $\subseteq_{f},\setminus_{f},\cup_{f},\cap_{f}$ will present the concept of subset, set difference, union, and intersection under fuzzy *s*-set environment.

**Definition** **13.** *A Δ-set $S_{\Delta }$ is defined as*

*$S_{\Delta }=\left\{\begin{array}{c} \left(\frac{\hat{b}}{\delta_{\Delta }\left(\hat{b}\right)},\vartheta_{\Delta }\left(\hat{b}\right)\right);\delta_{\Delta }\left(\hat{b}\right)\in F\left(\ddot{U}\right),\vartheta_{\Delta }\left(\hat{b}\right)\in F\left(\ddot{U}\right)\;\&\;\hat{b}\in B \end{array}\right\}$, where $\delta_{\Delta }:B\to I$ with $\delta_{\Delta }\left(\hat{b}\right)$ as fuzzy membership corresponds to each $\hat{b}\in B$ and $\vartheta_{\Delta }:B\to F\left(\ddot{U}\right)$ is a multiargument approximate function with $\vartheta_{\Delta }\left(\hat{b}\right)=\left\{\frac{{\hat{u}}_{i}}{\mu_{\hat{b}}\left({\hat{u}}_{i}\right)},{\hat{u}}_{i}\in \ddot{U}\right\}$ as fuzzy hypersoft approximate element of $S_{\Delta }$. The collection of all Δ-sets is denoted by $\Omega_{\Delta }$.*

The Δ-set is the generalization of *f*-set, *s*-set, fs-set, hs-set, fhs-set, and fpfs-set. Some of its particular cases are as follows:

1. It transforms to fpfs-set if hs-setting is replaced with *s*-setting.
2. It takes the form of fhs-set if fuzzy parameterization is omitted.
3. It converts to fs-set if fuzzy parameterization is ignored and hs-setting is replaced with *s*-setting.
4. It becomes *s*-set if fuzzy parameterization is ignored, hs-setting is replaced with *s*-setting and fuzzy grades are omitted.
5. It converts to *f*-set if fuzzy parameterization is ignored, hs-setting is replaced with *s*-setting, and fuzzy approximations are ignored.

**Example** **2.** *Let*$\ddot{U}=\left\{{\hat{u}}_{1},{\hat{u}}_{2},\ldots ,{\hat{u}}_{n}\right\}$*and*$E=\{{\ddot{\partial }}_{1},{\ddot{\partial }}_{2},\ldots ,{\ddot{\partial }}_{n}\}$. *The parameter-valued sets for each member of*E*are*$B_{1}=\left\{{\hat{b}}_{11},{\hat{b}}_{12}\right\}$*,*$B_{2}=\left\{{\hat{b}}_{21},{\hat{b}}_{22}\right\}$*, …,*$B_{n}=\left\{{\hat{b}}_{n1},{\hat{b}}_{n2}\right\}$*, and*$B=B_{1}*B_{2}*\ldots *B_{n}$*with*$B=\{{\hat{b}}_{1},{\hat{b}}_{1},\ldots ,{\hat{b}}_{k}\}$*, where each*${\hat{b}}_{i}$*is a n-tuple element of*B*with*$k=\prod_{i=1}^{n}\left|B_{i}\right|$*,*|.|*stands for set cardinality. The*Δ*-set*$S_{\Delta }$*can be constructed as*$S_{\Delta }=\left\{\begin{array}{c} \left(\frac{{\hat{b}}_{1}}{0.1},\left\{\frac{{\hat{u}}_{1}}{0.1},\frac{{\hat{u}}_{2}}{0.2}\right\}\right),\left(\frac{{\hat{b}}_{2}}{0.2},\left\{\frac{{\hat{u}}_{3}}{0.3},\frac{{\hat{u}}_{5}}{0.5}\right\}\right),\left(\frac{{\hat{b}}_{3}}{0.3},\left\{\frac{{\hat{u}}_{4}}{0.4},\frac{{\hat{u}}_{6}}{0.3}\right\}\right),\\ \ldots ,\left(\frac{{\hat{b}}_{k-1}}{0.5},\left\{\frac{{\hat{u}}_{2}}{0.2},\frac{{\hat{u}}_{n-2}}{0.5},\frac{{\hat{u}}_{n-1}}{0.1}\right\}\right),\left(\frac{{\hat{b}}_{k}}{0.6},\left\{\frac{{\hat{u}}_{2}}{0.2},\frac{{\hat{u}}_{n-1}}{0.4},\frac{{\hat{u}}_{n}}{0.3}\right\}\right) \end{array}\right\}$*, which states that for approximate element*$S_{\Delta }\left({\hat{b}}_{1}\right)$*with fuzzy membership*0.1*(i.e.,*10%*) in domain of*$S_{\Delta }$*, the alternatives*${\hat{u}}_{1}$*and*${\hat{u}}_{2}$*have fuzzy memberships*0.1*(i.e.,*10%*) and*0.2*(i.e.,*20%*), respectively. All remaining alternatives have fuzzy membership*0*corresponding to*${\hat{b}}_{1}\in B$*. Other approximate elements*$S_{\Delta }\left({\hat{b}}_{i}\right),i=2,3,\ldots ,n$*can be interpreted in a similar way.*

**Definition** **14.** *If $S_{\Delta }\in \Omega_{\Delta }$, then for $\delta_{\Delta }\left(\hat{b}\right)=0$ and $\vartheta_{\Delta }\left(\hat{b}\right)=\emptyset$ for all $\hat{b}\in B$, $S_{\Delta }$ is known as an empty Δ-set and is denoted by $S_{\emptyset }$.*

**Definition** **15.** *If $S_{\Delta }\in \Omega_{\Delta }$, then for $\delta_{\Delta }\left(\hat{b}\right)=1$ and $\vartheta_{\Delta }\left(\hat{b}\right)=\ddot{U}$ with fuzzy membership equal to 1 for all $\hat{b}\in B$, $S_{\Delta }$ is called a universal Δ-set and is denoted by $S_{\ddot{U}}$.*

**Definition** **16.** *Let $S_{\Delta_{1}},S_{\Delta_{2}}\in \Omega_{\Delta }$; then, $S_{\Delta_{1}}$ is a Δ-subset of $S_{\Delta_{2}}$, denoted by $S_{\Delta_{1}}\subseteq S_{\Delta_{2}}$ if $\delta_{\Delta_{1}}\left(\hat{b}\right)\leq \delta_{\Delta_{2}}\left(\hat{b}\right)$ and $\vartheta_{\Delta_{1}}\left(\hat{b}\right)\subseteq_{f}\vartheta_{\Delta_{2}}\left(\hat{b}\right)$ for all $\hat{b}\in B$.*

**Definition** **17.** *The Δ-set $S_{\Delta }^{c}$ is called the complement of $S_{\Delta }\in \Omega_{\Delta }$ if $\delta_{\Delta }^{c}\left(\hat{b}\right)=1-\delta_{\Delta }\left(\hat{b}\right)$ and $\vartheta_{\Delta }^{c}\left(\hat{b}\right)=\ddot{U}\setminus_{f}\vartheta_{\Delta }\left(\hat{b}\right)$ for all $\hat{b}\in B$.*

**Definition** **18.** *Let $S_{\Delta_{1}},S_{\Delta_{2}}\in \Omega_{\Delta }$; then, their union $S_{\Delta_{1}}\cup S_{\Delta_{2}}$ is a Δ-set $S_{\Delta }$ with $\delta_{\Delta }\left(\hat{b}\right)=max\left\{\delta_{\Delta_{1}}\left(\hat{b}\right),\delta_{\Delta_{2}}\left(\hat{b}\right)\right\}$ and $\vartheta_{\Delta }\left(\hat{b}\right)=\vartheta_{\Delta_{1}}\left(\hat{b}\right)\cup_{f}\vartheta_{\Delta_{2}}\left(\hat{b}\right)$ for all $\hat{b}\in B$.*

**Definition** **19.** *Let $S_{\Delta_{1}},S_{\Delta_{2}}\in \Omega_{\Delta }$; then, their intersection $S_{\Delta_{1}}\cup S_{\Delta_{2}}$ is a Δ-set $S_{\Delta }$ with $\delta_{\Delta }\left(\hat{b}\right)=min\left\{\delta_{\Delta_{1}}\left(\hat{b}\right),\delta_{\Delta_{2}}\left(\hat{b}\right)\right\}$ and $\vartheta_{\Delta }\left(\hat{b}\right)=\vartheta_{\Delta_{1}}\left(\hat{b}\right)\cap_{f}\vartheta_{\Delta_{2}}\left(\hat{b}\right)$ for all $\hat{b}\in B$.*

**Example** **3.** *Let $\ddot{U}=\left\{{\hat{u}}_{1},{\hat{u}}_{2},{\hat{u}}_{3},{\hat{u}}_{4},{\hat{u}}_{5}\right\}$ and $E=\{{\ddot{\partial }}_{1},{\ddot{\partial }}_{2},{\ddot{\partial }}_{3}\}$. The subparametric-valued disjoint sets corresponding to each member of E are $B_{1}=\left\{{\hat{b}}_{11}\right\},B_{2}=\left\{{\hat{b}}_{21},{\hat{b}}_{22}\right\}$, and $B_{3}=\left\{{\hat{b}}_{31},{\hat{b}}_{32}\right\}$, respectively; so, $B=B_{1}*B_{2}*B_{3}=\left\{{\hat{b}}_{1},{\hat{b}}_{2},{\hat{b}}_{3},{\hat{b}}_{4}\right\}$. The Δ-sets $S_{\Delta_{1}},S_{\Delta_{2}}$ are constructed as*

$S_{\Delta_{1}}=\left\{\begin{array}{c} \left(\frac{{\hat{b}}_{1}}{0.1},\left\{\frac{{\hat{u}}_{1}}{0.1},\frac{{\hat{u}}_{2}}{0.2}\right\}\right),\left(\frac{{\hat{b}}_{2}}{0.2},\left\{\frac{{\hat{u}}_{3}}{0.3},\frac{{\hat{u}}_{5}}{0.5}\right\}\right),\left(\frac{{\hat{b}}_{3}}{0.3},\left\{\frac{{\hat{u}}_{2}}{0.4},\frac{{\hat{u}}_{4}}{0.3}\right\}\right),\left(\frac{{\hat{b}}_{4}}{0.5},\left\{\frac{{\hat{u}}_{1}}{0.2},\frac{{\hat{u}}_{4}}{0.5}\right\}\right) \end{array}\right\}$

$S_{\Delta_{2}}=\left\{\begin{array}{c} \left(\frac{{\hat{b}}_{1}}{0.2},\left\{\frac{{\hat{u}}_{2}}{0.2},\frac{{\hat{u}}_{3}}{0.4}\right\}\right),\left(\frac{{\hat{b}}_{2}}{0.3},\left\{\frac{{\hat{u}}_{2}}{0.5},\frac{{\hat{u}}_{5}}{0.4}\right\}\right),\left(\frac{{\hat{b}}_{3}}{0.5},\left\{\frac{{\hat{u}}_{1}}{0.4},\frac{{\hat{u}}_{3}}{0.4}\right\}\right),\left(\frac{{\hat{b}}_{4}}{0.2},\left\{\frac{{\hat{u}}_{4}}{0.4},\frac{{\hat{u}}_{5}}{0.6}\right\}\right) \end{array}\right\}$

*then*

$S_{\Delta_{1}}\cup S_{\Delta_{2}}=\left\{\begin{array}{c} \left(\frac{{\hat{b}}_{1}}{0.2},\left\{\frac{{\hat{u}}_{1}}{0.1},\frac{{\hat{u}}_{2}}{0.2},\frac{{\hat{u}}_{3}}{0.4}\right\}\right),\left(\frac{{\hat{b}}_{2}}{0.3},\left\{\frac{{\hat{u}}_{2}}{0.5},\frac{{\hat{u}}_{3}}{0.3},\frac{{\hat{u}}_{5}}{0.5}\right\}\right),\\ \left(\frac{{\hat{b}}_{3}}{0.3},\left\{\frac{{\hat{u}}_{1}}{0.4},\frac{{\hat{u}}_{2}}{0.4},\frac{{\hat{u}}_{3}}{0.4},\frac{{\hat{u}}_{4}}{0.3}\right\}\right),\left(\frac{{\hat{b}}_{4}}{0.5},\left\{\frac{{\hat{u}}_{1}}{0.2},\frac{{\hat{u}}_{4}}{0.5},\frac{{\hat{u}}_{5}}{0.6}\right\}\right) \end{array}\right\}$

*and*

$S_{\Delta_{1}}\cap S_{\Delta_{2}}=\left\{\begin{array}{c} \left(\frac{{\hat{b}}_{1}}{0.1},\left\{\frac{{\hat{u}}_{2}}{0.2}\right\}\right),\left(\frac{{\hat{b}}_{2}}{0.2},\left\{\frac{{\hat{u}}_{5}}{0.4}\right\}\right),\left(\frac{{\hat{b}}_{3}}{0.3},\left\{\frac{\emptyset }{0.0}\right\}\right),\left(\frac{{\hat{b}}_{4}}{0.2},\left\{\frac{{\hat{u}}_{4}}{0.4}\right\}\right) \end{array}\right\}.$

## 4. Methodology and Algorithms

**Figure 1:**
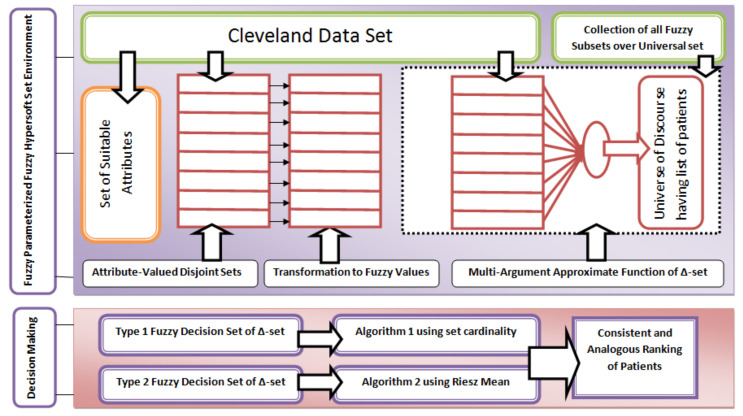
The pictographic demonstration of inclusive methodology.

In this section, an algorithm based on fuzzy decision set of Δ-set $S_{\Delta }$ is proposed for clinical DM by using CD-set [58]. The pictographic demonstration of the inclusive assumed methodology of the study is provided in Figure 1.

### 4.1. Aggregations of Δ-Set

**Definition** **20.** *Let Δ-set $S_{\Delta }\in \Omega_{\Delta }$; then, a type-1 fuzzy decision set of $S_{\Delta }$ (i.e., $S_{\Delta }^{D_{1}}$) is symbolized as $S_{\Delta }^{D_{1}}=\left\{\zeta_{\Delta }^{D_{1}}\left(\hat{u}\right)/\hat{u}:\hat{u}\in \ddot{U}\right\}$, where $\zeta_{\Delta }^{D_{1}}:\ddot{U}\to I$ and*

(3) $$\zeta_{\Delta }^{D_{1}}\left(\hat{u}\right)=\frac{1}{\left|B\right|}\sum_{\hat{b}\in B}\delta_{\Delta }\left(\hat{b}\right)\Gamma_{\vartheta_{\Delta }\left(\hat{b}\right)}\left(\hat{u}\right)$$

*where |B| stands for the set cardinality of B with*

(4) $$\Gamma_{\vartheta_{\Delta }\left(\hat{b}\right)}\left(\hat{u}\right)=\left\{\begin{array}{cc} \vartheta_{\Delta }\left(\hat{b}\right) & ;\;\;\;\;\;\hat{u}\in \Gamma_{\vartheta_{\Delta }}\left(\hat{b}\right)\\ 0 & ;\;\;\;\;\;\hat{u}\notin \Gamma_{\vartheta_{\Delta }}\left(\hat{b}\right) \end{array}\right..$$

*By Equation (3), it is observed that the following steps must be followed to compute the value of $\zeta_{\Delta }^{D_{1}}\left(\hat{u}\right)$:*

1. *Only select those parametric tuples that contain $\hat{u}$ in their approximations, i.e., the value of $\Gamma_{\vartheta_{\Delta }\left(\hat{b}\right)}\left(\hat{u}\right)$ will be equal to their corresponding fuzzy grades $\vartheta_{\Delta }\left(\hat{b}\right)$.*
2. *Compute the product of fuzzy parameterized value $\delta_{\Delta }\left(\hat{b}\right)$ and the obtained value of $\Gamma_{\vartheta_{\Delta }\left(\hat{b}\right)}\left(\hat{u}\right)$; then, determine the sum of these products.*
3. *Lastly, divide the computed sum with cardinality |B| of B.*

**Definition** **21.** *Let Δ-set $S_{\Delta }\in \Omega_{\Delta }$; then, a type-2 fuzzy decision set of $S_{\Delta }$ (i.e., $S_{\Delta }^{D_{2}}$) is symbolized as $S_{\Delta }^{D_{2}}=\left\{\zeta_{\Delta }^{D_{2}}\left(\hat{u}\right)/\hat{u}:\hat{u}\in \ddot{U}\right\}$, where $\zeta_{\Delta }^{D_{2}}:\ddot{U}\to I$ and*

(5) $$\zeta_{\Delta }^{D_{2}}\left(\hat{u}\right)=\frac{1}{X_{n}}\sum_{\hat{b}\in B}\delta_{\Delta }\left(\hat{b}\right)\Gamma_{\vartheta_{\Delta }\left(\hat{b}\right)}\left(\hat{u}\right)$$

*where $X_{n}$ stands for the value that is necessary to compute Riesz mean with*

(6) $$\Gamma_{\vartheta_{\Delta }\left(\hat{b}\right)}\left(\hat{u}\right)=\left\{\begin{array}{cc} \vartheta_{\Delta }\left(\hat{b}\right) & ;\;\;\;\;\;\hat{u}\in \Gamma_{\vartheta_{\Delta }}\left(\hat{b}\right)\\ 0 & ;\;\;\;\;\;\hat{u}\notin \Gamma_{\vartheta_{\Delta }}\left(\hat{b}\right) \end{array}\right..$$

*Similarly, by Equation (5), the first two steps are the same as in Definition 20 to compute the value of $\zeta_{\Delta }^{D_{2}}\left(\hat{u}\right)$; however, the third step is given as follows:*

- *Divide the computed sum with the value $X_{n}$ that is explained in Definition 12 and Example 1.*

### 4.2. Cleveland Dataset

The CD-set [58] was developed for the analytical study of heart ailments. From the CD-set, a total three hundred and three patients were examined for the identification of heart-based ailments by taking into account seventy-six traits (nevertheless, only fourteen can be utilized for experimentation and investigation) with five outcomes. The depiction of these fourteen traits is tabulated in Table 1. In order to justify and implement hs-setting, six patients were selected to be examined for heart-based ailments by assuming nine of the most fitting traits. The portrayal view of these traits in conjunction with their CD-set-based values is presented in Table 2.

### 4.3. Salient Features of Opted Attributes

**Figure 2:**
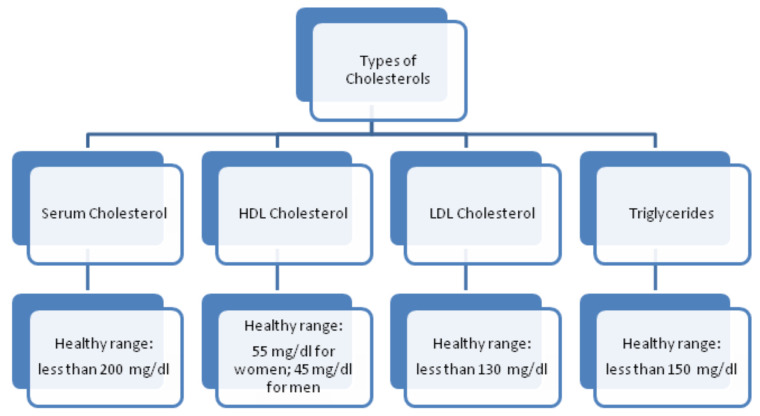
Types of Cholesterol and their healthy ranges.

**Figure 3:**
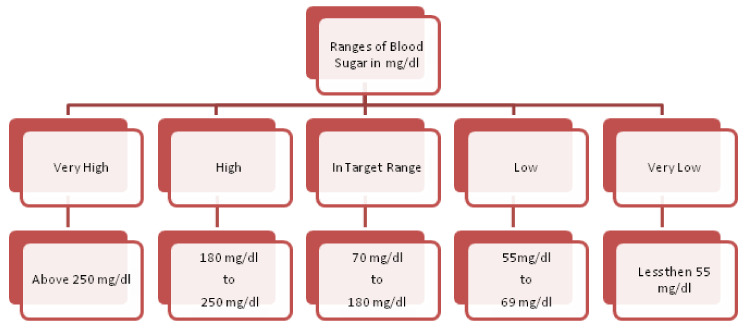
Ranges of Blood Sugar.

**Figure 4:**
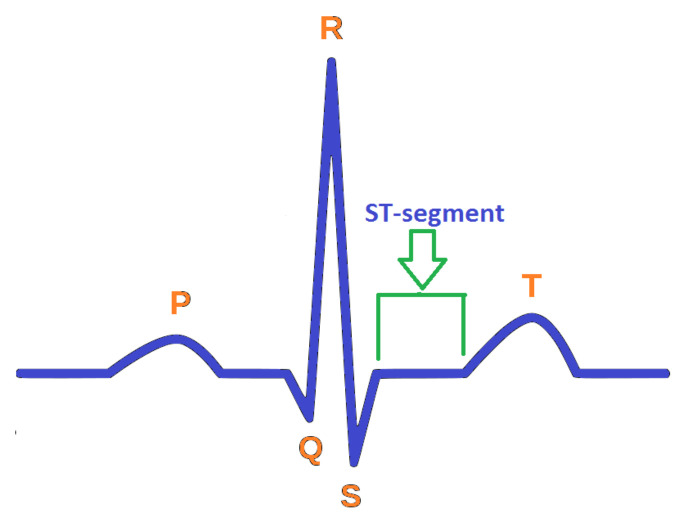
ST-segment in ECG (source: Wikipedia).

**Figure 5:**
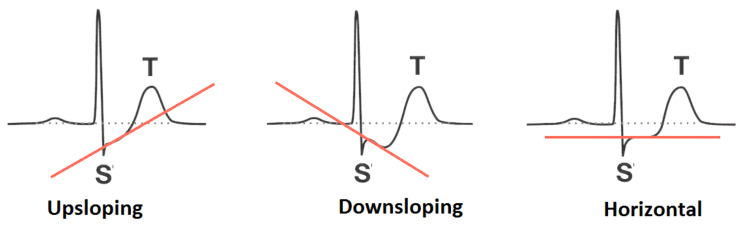
Pictographic view of ST-segment (source: https://litfl.com/st-segment-ecg-library (accessed on 3 October 2021)).

In order to have justification regarding the selection of attributes, this segment describes some of their salient features for heart-based ailments analysis. The features are conferred underneath:

1. **Age**. Aging is a self-determining menace aspect for heart ailments. Although this factor is reported higher in aged persons (more than 60 years), with the involvement of various supplementary reasons, adults can also be in danger. The cardiologists have classified the aging factor into four groups: (i) 20 years or less, (ii) 40 years or less, (iii) 60 years or less, (iv) more than 60 years.
2. **Chest Pain Type**. Chest pain is a significant factor that leads to the suffering of cardiac disorder. It may vary due to quality, span, area, and force. Its intensity may be sharp, distressing feeling, and deadly upset. The chest pain attached with heart diseases can be sorted as Typical Angina (TA), Atypical Angina (ATA), Non-Anginal pain, and Asymptomatic (AM) (see [58]). The first two types are considered significant factors towards the suffering of heart diseases; the others are of less significance but cannot be ignored.
3. **Resting Blood Pressure**. This pressure is produced due to blood flow in blood vessels on its walls. The narrowness of the blood vessels is reported due to this pressure. The medical experts have sorted it as systolic and diastolic. These are produced during active blood flow and relaxing state, respectively. Its measuring unit is mm Hg, in accordance with dataset. The standard values for systolic and diastolic are 120 and 80 mm Hg, respectively. More than 120 mm Hg and less than 80 mm Hg (see [59]) are considered abnormal values for systolic and diastolic, respectively.
4. **Serum Cholesterol**. Cholesterol is a variety of fat, recognized as lipid, which is encapsulated in proteins bundles (lipoproteins) and flows in blood vessels and capillaries. The common types of cholesterol are LDL, HDL, and triglycerides. These cholesterols cause the narrowness of the blood vessels, which may lead to severe heart issue. The LDL and HDL are also regarded as bad cholesterol and good cholesterol, respectively. A particular lab test ”Lipid Profile Test (LPT)“ is used to assess the values of these cholesterols. Its measuring unit is mg/dL, which is used in the adopted dataset. The serum cholesterol depends upon these cholesterol collectively and its level is determined by summing up the values of HDL and LDL along with 20% of triglycerides. Its values lie in the interval [126, 564] (see [60]). The types of cholesterol and their ranges are provided in Figure 2.
5. **Fasting Blood Sugar**. This is regarded as another authentic factor for the analysis of heart diseases. It is usually observed that heart patients have high glucose due to the ”tension reaction“. In other words, nondiabetic patients may also have its high ratio. The ranges for its usual observed values are presented in Figure 3. Its measuring unit is mg/dL, which is used in the adopted dataset. A value of 120 mg/dL (see [58]) is regarded as a typical value for healthy individual.
6. **Maximum Heart Rate Achieved**. Heart rate is the number of hearts beats per minute (bpm) and is regarded as a reliable source to determine the oxygen utilization in heart patients. Its values lies in the interval of 71 bpm, 195 bpm (see [61]).
7. **Oldpeak and Slope**. Oldpeak is usually meant for Shock-Toxicity depression (also known as ST-depression), which is provoked by rest-base work out. It is regarded as a trustworthy ECG (electrocardiogram) result for the analysis of disruptive coronary issues. Its measuring unit is mm, which can take values from the interval [0.0, 0.5]. Figure 4 presents its pictographic view. Its slope can be sorted into three types (see [58]): (i) Upsloping, (ii) Flat (Horizontal), (iii) Downsloping. The pictorial display of these categories is presented in Figure 5.
8. **Thal**. This is a familiar turmoil of blood recognized as thalassemia, which can be sorted into four categories: (i) Null (i.e., no flow of blood at all) (ii) Fixed Defect (i.e., partial flow of blood in some sections of the heart), (iii) Normal Blood Flow, and (iv) Reversible Defect (i.e., observation of blood flow without normality). The corresponding values assigned by medical experts to these categories are 0, 3, 6, and 7, respectively (see [58]). In case of heart disease diagnosis, the category (i) is usually disregarded.

### 4.4. Determination of Fuzzy-Values-Based Ranges for Opted Parameters

This part aims to describe a criterion to convert the original values (the allocated valued by CD-set) of parameters to fuzzy-values-based ranges. This task is accomplished with the employment of an algebraic criterion whereby its fuzzy-values-based range with respect to each parameter is determined by dividing its allocated values with maximum allocated value. For example, in Table 3, the maximum value is 80 years against the first parameter; therefore, by dividing all other allocated values of age by 80, the required ranges are obtained. In this way, the fuzzy-values-based ranges for remaining parameters are determined. Table 3 presents all such ranges.

### 4.5. Declaration of Problem

Mathematical approaches for medical identification of definite ailments have earned immense concentration from scholars. These approaches may entail factual or imaginary information/records. With the introduction of *f*-set, investigators have been tempted to *f*-set-based approaches for medicinal analysis with vague settings. Several developments have been established in this field. The fhs-set has gained much significance in this regard as it has the potential to generalize the classical models and to manage the shortcomings depicted by these structures. It is scrutinized that few researches have been reported so far relating to medicinal study of definite ailments based on mathematical context with fhs-setting and fuzzy parameterization setting by assuming factual data. It is a commendable aspect of this study that factual variables of CD-set have been utilized to the context of medicinal analysis of heart-related ailments under a reliable-cum-flexible model. The factual input variables are assigned a specific degree of uncertainty to assist the medical expert in judging the vague nature of these variables.

### 4.6. Proposed Algorithm Based on Δ-set and Its Implementation

Now, an algorithm (Algorithm 1) is put forward by taking into consideration the aggregations of Δ-set with the aim of medicinal identification of heart-related diseases.
**Algorithm 1:** Steps for the analysis of heart-related diseases based on Δ-set.▹**Start**▹**Input:**1. Assume $\ddot{U}=\left\{{\hat{p}}_{1},{\hat{p}}_{2},{\hat{p}}_{3},\ldots ,{\hat{p}}_{k}\right\}$ as an initial universe containing the list of patients being examined.2. Assume $\hat{E}=\left\{{\ddot{\partial }}_{1},{\ddot{\partial }}_{2},{\ddot{\partial }}_{3},\ldots ,{\ddot{\partial }}_{n}\right\}$ as a collection of attributes.3. Categorize the elements of $\hat{E}$ into nonoverlapping subclasses containing their subparametric values:${\hat{E}}^{1}=\left\{{\ddot{\partial }}^{11},{\ddot{\partial }}^{12},\ldots ,{\ddot{\partial }}^{1n}\right\},{\hat{E}}^{2}=\left\{{\ddot{\partial }}^{21},{\ddot{\partial }}^{22},\ldots ,{\ddot{\partial }}^{2n}\right\},{\hat{E}}^{3}=\left\{{\ddot{\partial }}^{31},{\ddot{\partial }}^{32},\ldots ,{\ddot{\partial }}^{3n}\right\},\ldots ,\;{\hat{E}}^{n}=\left\{{\ddot{\partial }}^{n1},{\ddot{\partial }}^{n2},\ldots ,{\ddot{\partial }}^{nn}\right\}.$▹**Construction:**4. Determine C-product $G={\hat{E}}^{1}*{\hat{E}}^{2}*{\hat{E}}^{3}*\ldots *{\hat{E}}^{n}=\left\{{\ddot{℘}}_{1},{\ddot{℘}}_{2},{\ddot{℘}}_{3},\ldots ,{\ddot{℘}}_{r}\right\}$ with $r=\prod_{i=1}^{n}\left|{\hat{E}}^{i}\right|$, where $|{\hat{E}}^{i}|$ stands for the cardinality of sets ${\hat{E}}^{i}$.5. Take $H=\{{\ddot{℘}}_{1},{\ddot{℘}}_{2},{\ddot{℘}}_{3},\ldots ,{\ddot{℘}}_{s}\}\subseteq G$ such that s≤r on the basis of decision-makers consultation.6. Construct Δ-set $S_{\Delta }$ by using Definition 13 and represent it in tabular notation.▹**Computation:**7. Compute $\zeta_{\Delta }^{D}$ corresponding to each element ${\hat{p}}_{i},i=1,2,\ldots ,k$ of $\ddot{U}$ by using Definition 20.8. Compute $S_{\Delta }^{D}$ by using Definition 20.▹**Output:**9. Choose the Max$\left\{\zeta_{\Delta }^{D}\left({\hat{p}}_{i}\right)\right\}$ as final selection.▹**End**

**Figure 6:**
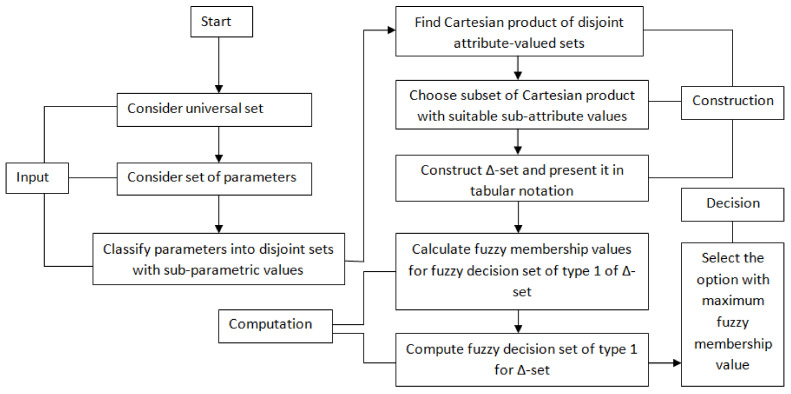
Algorithm Based on Decision Set of Type-1.

The procedural flow of this algorithm is displayed in Figure 6.

Now, the above algorithm is elaborated with the application below.

**Example** **4.** ***Input Stage: (Step 1–Step 3)** In order to make the computations easy, 6 patients are considered who are likely to be examined for heart-related disease. The initial universe $\ddot{U}=\left\{{\hat{p}}_{1},{\hat{p}}_{2},{\hat{p}}_{24},{\hat{p}}_{25},{\hat{p}}_{75},{\hat{p}}_{303}\right\}$ is constructed. Suppose that $\hat{E}=\left\{{\ddot{\partial }}_{1},{\ddot{\partial }}_{2},{\ddot{\partial }}_{3},{\ddot{\partial }}_{4},{\ddot{\partial }}_{5},{\ddot{\partial }}_{6},{\ddot{\partial }}_{7},{\ddot{\partial }}_{8},{\ddot{\partial }}_{9}\right\}$ is the collection of parameters with descriptions such as ${\ddot{\partial }}_{1}=$ age, ${\ddot{\partial }}_{2}=$ chest pain type, ${\ddot{\partial }}_{3}=$ resting blood pressure, ${\ddot{\partial }}_{4}=$ serum cholesterol, ${\ddot{\partial }}_{5}=$ fasting blood sugar, ${\ddot{\partial }}_{6}=$ maximum heart rate achieved, ${\ddot{\partial }}_{7}=$ old peak, ${\ddot{\partial }}_{8}=$ slope, and ${\ddot{\partial }}_{9}=$ thal. Their parametric-valued subclasses are as follows:*

${\hat{E}}^{1}=\left\{\begin{array}{c} {\ddot{\partial }}^{11}=category1,{\ddot{\partial }}^{12}=category2,\\ {\ddot{\partial }}^{13}=category3,{\ddot{\partial }}^{14}=category4 \end{array}\right\},$

${\hat{E}}^{2}=\left\{\begin{array}{c} {\ddot{\partial }}^{21}=typicalangina,{\ddot{\partial }}^{22}=atypicalangina,\\ {\ddot{\partial }}^{23}=non-anginalpain,{\ddot{\partial }}^{24}=asymptomatic \end{array}\right\},$

${\hat{E}}^{3}=\left\{\begin{array}{c} {\ddot{\partial }}^{31}=110\;\mathrm{mmHg},{\ddot{\partial }}^{32}=150\;\mathrm{mmHg},\\ {\ddot{\partial }}^{33}=180\;\mathrm{mmHg} \end{array}\right\},$

${\hat{E}}^{4}=\left\{\begin{array}{c} {\ddot{\partial }}^{41}=210\;\mathrm{mg}/\mathrm{dL},{\ddot{\partial }}^{42}=320\;\mathrm{mg}/\mathrm{dL},\\ {\ddot{\partial }}^{43}=430\;\mathrm{mg}/\mathrm{dL} \end{array}\right\},$

${\hat{E}}^{5}=\left\{\begin{array}{c} {\ddot{\partial }}^{51}=120\;\mathrm{mg}/\mathrm{dL} \end{array}\right\},$

${\hat{E}}^{6}=\left\{\begin{array}{c} {\ddot{\partial }}^{61}=81,{\ddot{\partial }}^{62}=140 \end{array}\right\},$

${\hat{E}}^{7}=\left\{\begin{array}{c} {\ddot{\partial }}^{71}=1.2,{\ddot{\partial }}^{72}=3.7 \end{array}\right\},$

${\hat{E}}^{8}=\left\{\begin{array}{c} {\ddot{\partial }}^{81}=upsloping,{\ddot{\partial }}^{82}=flat,\\ {\ddot{\partial }}^{83}=downsloping \end{array}\right\},$

${\hat{E}}^{9}=\left\{\begin{array}{c} {\ddot{\partial }}^{91}=normal,{\ddot{\partial }}^{92}=fixeddefect,\\ {\ddot{\partial }}^{93}=reversibledefect \end{array}\right\}.$

***Construction Stage: Step 4***

*In this step, compute the C-product $G={\hat{E}}^{1}*{\hat{E}}^{2}*{\hat{E}}^{3}*\ldots *{\hat{E}}^{9}=\left\{{\ddot{℘}}_{1},{\ddot{℘}}_{2},{\ddot{℘}}_{3},\ldots ,{\ddot{℘}}_{r}\right\}$, where r is the product of cardinalities of ${\hat{E}}^{i}$.*

***Step 5:***

*With the mutual understanding and consensus of medical experts, ${\ddot{\partial }}^{12}$ and ${\ddot{\partial }}^{13}$ are given preference in ${\hat{E}}^{1}$, ${\ddot{\partial }}^{21}$ and ${\ddot{\partial }}^{22}$ in ${\hat{E}}^{2}$, ${\ddot{\partial }}^{32}$ in ${\hat{E}}^{3}$, ${\ddot{\partial }}^{42}$ in ${\hat{E}}^{4}$, ${\ddot{\partial }}^{51}$ in ${\hat{E}}^{5}$, ${\ddot{\partial }}^{61}$ and ${\ddot{\partial }}^{62}$ in ${\hat{E}}^{6}$, ${\ddot{\partial }}^{72}$ in ${\hat{E}}^{7}$, ${\ddot{\partial }}^{83}$ in ${\hat{E}}^{8}$, and ${\ddot{\partial }}^{92}$ in ${\hat{E}}^{9}$. Thus, the set $H=\{{\ddot{\hbar }}_{1},{\ddot{\hbar }}_{2},{\ddot{\hbar }}_{3},{\ddot{\hbar }}_{4},{\ddot{\hbar }}_{5},{\ddot{\hbar }}_{6},{\ddot{\hbar }}_{7},{\ddot{\hbar }}_{8}\}$ is constructed.*

***Step 6:***

*Now, we calculate fuzzy membership values $\mu ({\ddot{\partial }}^{ij})$ and $\mu ({\ddot{\hbar }}_{i})$ in accordance with Figure 3 for each ${\ddot{\partial }}^{ij}$ and ${\ddot{\hbar }}_{i}$, respectively, preferred by medical specialist. The fuzzy membership $\mu ({\ddot{\hbar }}_{1})$ of ${\ddot{\hbar }}_{1}$ is equal to the arithmetic mean of the fuzzy membership values of ${\ddot{\partial }}^{ij}$ belonging to tuple ${\ddot{\hbar }}_{1}$. Similarly, the fuzzy membership values of the remaining ${\ddot{\hbar }}^{i},i=2,3,\ldots ,8$ can be calculated in the same manner. These calculated values are given in Table 4 and Table 5, respectively.*

*Now, we construct Δ-set $S_{\Delta }$ by using Definition 13*

$$S_{\Delta }=\left\{\begin{array}{c} \left(\frac{{\ddot{\hbar }}_{1}}{0.667},\left\{\frac{{\hat{p}}_{1}}{0.2},\frac{{\hat{p}}_{2}}{0.3},\frac{{\hat{p}}_{24}}{0.0},\frac{{\hat{p}}_{25}}{0.4},\frac{{\hat{p}}_{75}}{0.6},\frac{{\hat{p}}_{303}}{0.7}\right\}\right),\left(\frac{{\ddot{\hbar }}_{2}}{0.701},\left\{\frac{{\hat{p}}_{1}}{0.0},\frac{{\hat{p}}_{2}}{0.4},\frac{{\hat{p}}_{24}}{0.5},\frac{{\hat{p}}_{25}}{0.6},\frac{{\hat{p}}_{75}}{0.7},\frac{{\hat{p}}_{303}}{0.8}\right\}\right),\\ \left(\frac{{\ddot{\hbar }}_{3}}{0.695},\left\{\frac{{\hat{p}}_{1}}{0.3},\frac{{\hat{p}}_{2}}{0.5},\frac{{\hat{p}}_{24}}{0.3},\frac{{\hat{p}}_{25}}{0.0},\frac{{\hat{p}}_{75}}{0.4},\frac{{\hat{p}}_{303}}{0.5}\right\}\right),\left(\frac{{\ddot{\hbar }}_{4}}{0.729},\left\{\frac{{\hat{p}}_{1}}{0.5},\frac{{\hat{p}}_{2}}{0.4},\frac{{\hat{p}}_{24}}{0.3},\frac{{\hat{p}}_{25}}{0.2},\frac{{\hat{p}}_{75}}{0.0},\frac{{\hat{p}}_{303}}{0.1}\right\}\right),\\ \left(\frac{{\ddot{\hbar }}_{5}}{0.690},\left\{\frac{{\hat{p}}_{1}}{0.0},\frac{{\hat{p}}_{2}}{0.2},\frac{{\hat{p}}_{24}}{0.3},\frac{{\hat{p}}_{25}}{0.4},\frac{{\hat{p}}_{75}}{0.5},\frac{{\hat{p}}_{303}}{0.6}\right\}\right),\left(\frac{{\ddot{\hbar }}_{6}}{0.723},\left\{\frac{{\hat{p}}_{1}}{0.4},\frac{{\hat{p}}_{2}}{0.4},\frac{{\hat{p}}_{24}}{0.5},\frac{{\hat{p}}_{25}}{0.6},\frac{{\hat{p}}_{75}}{0.8},\frac{{\hat{p}}_{303}}{0.0}\right\}\right),\\ \left(\frac{{\ddot{\hbar }}_{7}}{0.717},\left\{\frac{{\hat{p}}_{1}}{0.3},\frac{{\hat{p}}_{2}}{0.6},\frac{{\hat{p}}_{24}}{0.4},\frac{{\hat{p}}_{25}}{0.4},\frac{{\hat{p}}_{75}}{0.5},\frac{{\hat{p}}_{303}}{0.2}\right\}\right),\left(\frac{{\ddot{\hbar }}_{8}}{0.751},\left\{\frac{{\hat{p}}_{1}}{0.7},\frac{{\hat{p}}_{2}}{0.5},\frac{{\hat{p}}_{24}}{0.3},\frac{{\hat{p}}_{25}}{0.5},\frac{{\hat{p}}_{75}}{0.4},\frac{{\hat{p}}_{303}}{0.3}\right\}\right) \end{array}\right\}.$$

**Figure 7:**
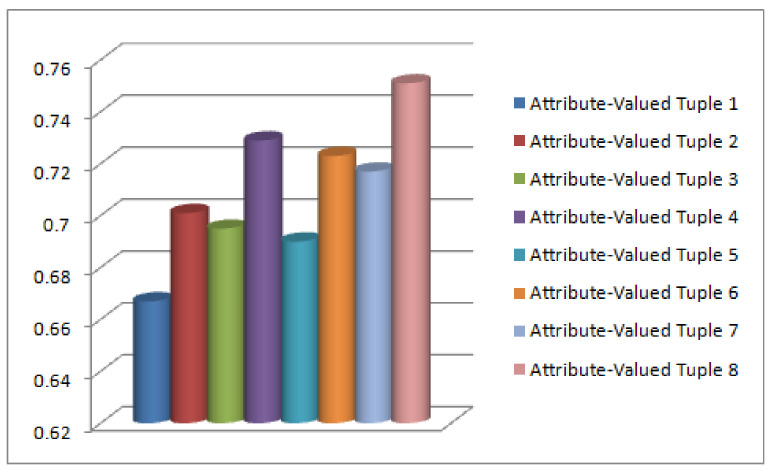
Fuzzy Membership Values corresponding to ${\ddot{\hbar }}_{i}$.

*Its tabular representation is given in Table 6 (see Figure 7 for graphical interpretation).*

***Computation Stage: Step 7***

*Now, we calculate fuzzy membership $\zeta_{\Delta }^{D_{1}}$ of fuzzy decision set $S_{\Delta }^{D_{1}}$ for Δ-set $S_{\Delta }$ corresponding to each ${\hat{p}}_{i}$. For this purpose, we need to find the containment status of each ${\hat{p}}_{i}$ in approximate values of ${\ddot{\hbar }}_{i}$. Such information is given in Table 7 and, with the help of this information, fuzzy membership $\zeta_{\Delta }^{D_{1}}$ is computed for each ${\hat{p}}_{i}$ and given in Table 8.*

***Step 8:***

*Now, we construct fuzzy decision set $S_{\Delta }^{D_{1}}$ for Δ-set $S_{\Delta }$ corresponding to all ${\hat{p}}_{i}$ by using their fuzzy membership values $\zeta_{\Delta }^{D_{1}}\left({\hat{p}}_{i}\right)$, which are given in Table 8.*

**Figure 8:**
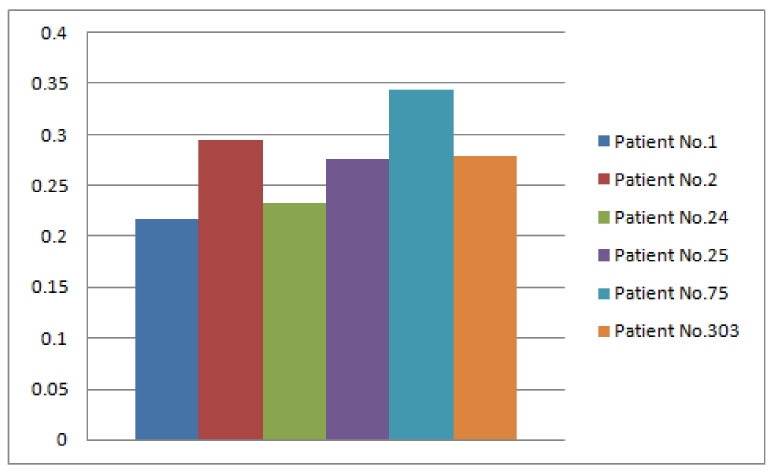
Fuzzy membership $\zeta_{\Delta }^{D_{1}}$ for each ${\hat{p}}_{i}$.

*The values of Table 8 are interpreted graphically in Figure 8.*

*$S_{\Delta }^{D_{1}}=\left\{\begin{array}{c} 0.217050/{\hat{p}}_{1},0.294063/{\hat{p}}_{2},0.232288/{\hat{p}}_{24},\\ 0.275663/{\hat{p}}_{25},0.343900/{\hat{p}}_{75},0.278850/{\hat{p}}_{303} \end{array}\right\}$.*

***Decision Stage: Step 9***

*The maximum value of $\zeta_{\Delta }^{D_{1}}\left({\hat{p}}_{i}\right)$ is 0.343900 for ${\hat{p}}_{75}$. Hence, it is observed that the patient ${\hat{p}}_{75}$ is expected to be diagnosed for heart disease.*

### 4.7. Proposed Algorithm Based on Riesz Summability

In this part of the paper, another algorithm (Algorithm 2) is put forward by taking into consideration the concept of Riesz Summability to diagnose heart-related diseases in patients.
**Algorithm 2:** Analysis of Heart-related Diseases through the concept of Riesz Summability.▹**Start**▹**Input:** (1.–3.) Same as in Algorithm 1.▹**Construction:** (4.–6.) Same as in Algorithm 1.▹**Computation:**7. Compute $X_{n}=\sum_{i=1}^{n}{\hat{x}}_{i}$ for the determination of Riesz mean according to Definition 12.8. Compute $\zeta_{\Delta }^{D_{2}}$ for all ${\hat{p}}_{i}$ in accordance with Definition 21.9. Compute $S_{\Delta }^{D_{2}}$ by using Definition 21.▹**Output:**10. Choose the Max$\left\{\zeta_{\Delta }^{D_{2}}\left({\hat{p}}_{i}\right)\right\}$ as final selection.▹**End**

**Figure 9:**
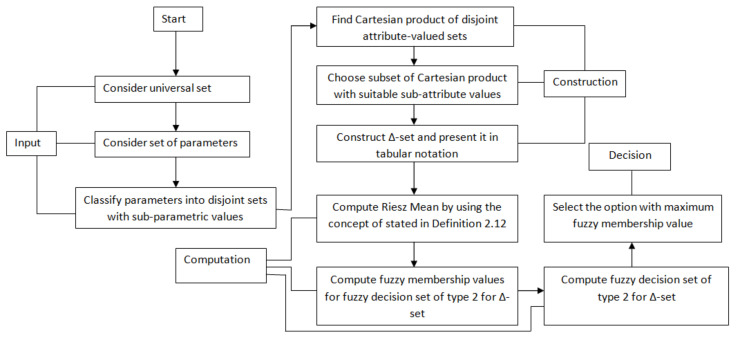
Algorithm Based on Decision Set of Type-2.

The procedural flow of this algorithm is displayed in Figure 9.

The above algorithm is validated with the help of the following example.

**Example** **5.** *Consider the data from Example 4, which covers all the steps of first two stages, i.e., input stage and construction stage of Algorithm 2. Therefore, we start with computation stage as given below.*

***Computation: Step 7***

*Let $\mu \left({\ddot{\hbar }}_{1}\right)={\hat{x}}_{1},\mu \left({\ddot{\hbar }}_{2}\right)={\hat{x}}_{2},\mu \left({\ddot{\hbar }}_{3}\right)={\hat{x}}_{3},\mu \left({\ddot{\hbar }}_{4}\right)={\hat{x}}_{4},\mu \left({\ddot{\hbar }}_{5}\right)={\hat{x}}_{5},\mu \left({\ddot{\hbar }}_{6}\right)={\hat{x}}_{6},\mu \left({\ddot{\hbar }}_{7}\right)={\hat{x}}_{7}$ and $\mu \left({\ddot{\hbar }}_{8}\right)={\hat{x}}_{8}$. Then $X_{8}=\sum_{i=1}^{8}{\hat{x}}_{i}=0.667+0.701+0.695+0.729+0.690+0.723+0.717+0.751=5.673$.*

***Step 8:***

*Now, we calculate fuzzy membership $\zeta_{\Delta }^{D_{2}}$ for each ${\hat{p}}_{i}$ by using Definition 21, Table 6 and Table 7. The calculated fuzzy membership values are given in Table 9.*

**Figure 10:**
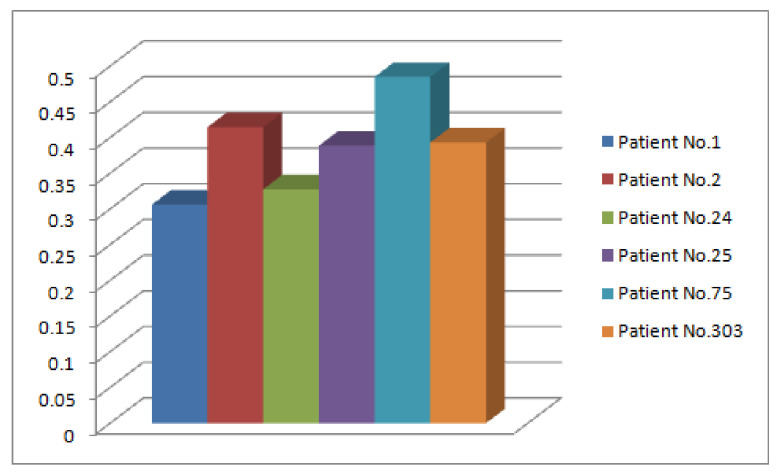
Fuzzy membership $\zeta_{\Delta }^{D_{2}}$ for each ${\hat{p}}_{i}$.

*The graphical interpretation of Table 9 is presented in Figure 10.*

***Step 9:***

*Now, we construct fuzzy decision set $S_{\Delta }^{D_{2}}$ for Δ-set $S_{\Delta }$ corresponding to all ${\hat{p}}_{i}$ by using their fuzzy membership values $\zeta_{\Delta }^{D_{2}}\left({\hat{p}}_{i}\right)$, which are given in Table 9.*

*$S_{\Delta }^{D_{2}}=\left\{\begin{array}{c} 0.306081/{\hat{p}}_{1},0.414684/{\hat{p}}_{2},0.327569/{\hat{p}}_{24},\\ 0.388736/{\hat{p}}_{25},0.484964/{\hat{p}}_{75},0.393231/{\hat{p}}_{303} \end{array}\right\}$.*

***Decision Stage: Step 10***

*As the maximum value of $\zeta_{\Delta }^{D_{2}}\left({\hat{p}}_{i}\right)$ is 0.484964 for ${\hat{p}}_{75}$, it is observed that the patient ${\hat{p}}_{75}$ is expected to be diagnosed for heart disease.*

**Figure 11:**
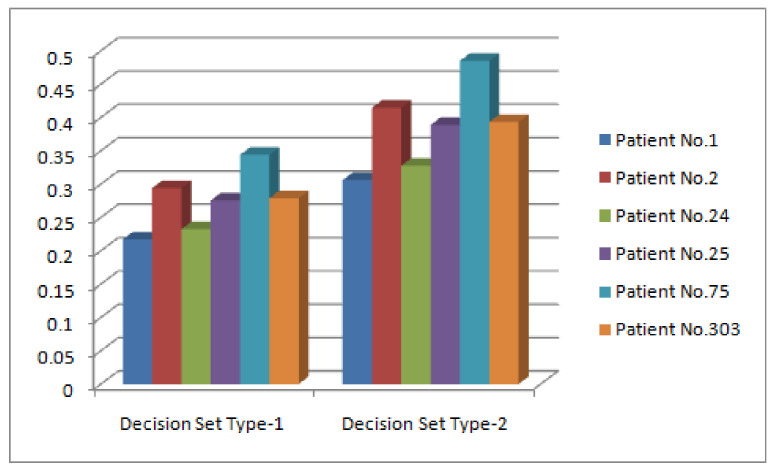
Comparison of Decision Sets of Type-1 and Type-2.

*The comparison of the results obtained from both algorithms is presented in Figure 11.*

## 5. Discussion and Comparison Analysis

Yılmaz et al. [40] applied and compared the concepts of fpfs-set and Riesz Summability given by Çağman et al. [39] and Altay et al. [55], respectively, for solving decision-making problem with hypothetical data under uncertain environment. Kirişci [41,42] employed CD-set for the diagnosis of heart diseases through decision-making techniques based fs-set. Rahman et al. [48] conceptualized Δ-set as a generalization of fs-set [3] and fpfs-set [39,43,44]. This study employed fuzzy decision set techniques (modification of aggregations discussed in [39]) of Δ-set for solving medical decision-making problem with real values of attributes from CD-set. Kirişci used single-argument approximate function of fs-set to deal with 11 attributes out of 14 prescribed attributes from CD-set. He assigned hypothetical fuzzy membership values to these fuzzy parameters without any appropriate criterion. As the single-argument approximate function maps attributes to subsets of universal set, subparametric values of adopted attributes are not focused and ignored, which raises questions as to the reliability of decision-making. In short, the above mentioned existing models are not capable to manage the following settings collectively:

1. The setting when parameters and their subparametric-values-based tuples are ambiguous, i.e., decision makers are not sure about their preference-based selection. In other words, the parameters and their subparametric-values-based tuples are uncertain for decision-makers.
2. The setting where it is necessary to categorize the parameters into their related disjoint subclasses having their subparametric values. This setting demands the entitlement of multiargument approximate function, which has the capability to cope with such subparametric-valued disjoint classes. Its domain is the C-product of these classes and range is the subsets of initial universe.

**Figure 12:**
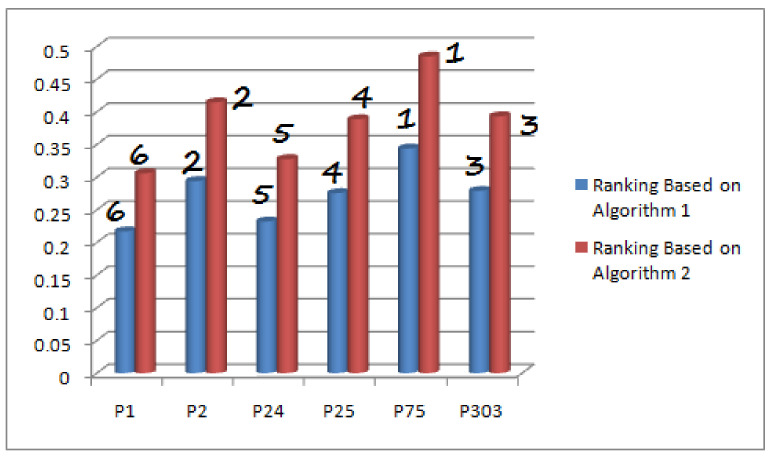
Ranking Comparison of Both Proposed Algorithms.

On the contrary, this study has used the multiargument approximate function, which not only focuses on attributes but also emphasizes on their corresponding attributive values. The real attributes are taken from CD-set and then these values are converted to their related fuzzy values by employing an appropriate criterion rather than assigning hypothetical values. The selected attributes are further partitioned into disjoint sets having their respective subattributive values. The C-product of these sets is obtained to furnish the requirement for the domain of multiargument approximate function. Each element of this domain is further transformed to fuzzy grades to cope with the scenario of Δ-set. Two types of fuzzy decision sets are introduced for Δ-set on the basis of set cardinality and Riesz mean that have further been used to propose algorithms for solving medical decision-making problem for the diagnosis of heart diseases. The results have been compared and found successful. It has been observed that both algorithms yield different fuzzy membership values for patients under consideration but provided the same rankings (see Figure 12). The problem of heart-disease-based medical diagnosis has not been addressed by any author in literature under fuzzy parameterized-like models. Therefore, numerical-results-based comparison of the proposed study is not possible with any existing fuzzy-set-like models; however, its structural comparison is discussed with most relevant models to assess the flexibility and advantageous aspects. Table 10 and Table 11 present the structural comparison of the proposed study by taking into consideration few pertinent factors.

### Merits of Proposed Study

Now, some merits of this study are underlined as follows:

1. The presented approach took the importance of inspiration of fuzzy-parameterization associated by Δ-set to manage modern-day DM issues. The assignment of parameterized fuzzy grade imitates the possibility of recognition level; in this way, it has incredible prospective in the real description within the scope of computational scenarios.
2. Real attributes of CD-set are converted to fuzzy membership by using algebraic technique.
3. The sequential nature of approximate values of Δ-set is managed by employing classical concept of Riesz Summability and analogous results have been achieved.
4. Since the presented model put emphasis on comprehensive study of parameters (i.e., additional classification of parameters) more willingly than focusing on parameters merely, consequently, it enables decision-makers to have better and more reliable decisions.
5. The two proposed algorithms have ranked the patients with analogous and consistent results by considering a smaller number of attributes.

## 6. Conclusions

In this article, a multiattribute, decision-based medical diagnosis for heart diseases is discussed by using two set-theoretic models, i.e., Δ-set and Riesz Summability. The former one Δ-set is the generalization of fuzzy parameterized fuzzy soft set, fuzzy parameterized soft set, fuzzy soft set, and soft set. It is capable of managing the shortcomings of such structures regarding deliberation of approximate mapping with multiarguments. This kind of mapping considers the C-product of subparametric tuples as its domain and then maps them to the power set of universal set. It lays emphasis on the classification of each parameter into its respective parametric-valued sets, which is not considered by existing soft-set-like models. The later one is a classical approach of mathematical analysis, which is projected to tackle the sequential nature of uncertain data. As it is commonly observed that data used in medical diagnosis are of sequential and uncertain nature, both issues are resolved by using Δ-set and Riesz Summability. The input variables are taken from CD-set, and the operational role of each variable is investigated. Factual input values are converted to relevant fuzzy membership values. Two algorithms based on two types of decision set for Δ-set are proposed and validated with examples for diagnosis of patients for heart diseases. Both algorithms are proved consistent and analogous results are achieved. As this study has considered only fuzzy membership for dealing with uncertainties in parameters as well as fuzzy hypersoft numbers, it depicts inadequacy to tackle scenarios with entitlement of nonmembership and indeterminacy grades. Therefore, it can be extended to manage such scenarios. Moreover, this can further be studied by discussing other cases under vague settings with fuzzy parameterized settings by using more than nine attributes and more than six patients. Its scope covers a wide range of computational intelligence and neuroscience under fuzzy-set-like environments.

## Figures and Tables

**Table 1:** Brief description of parameters in CD-set.

Ordering by Scrutiny	Ordering by CD-Set	Parameters (Short Names)	Parameters (Full Names)
1	3	age	Age in years
2	4	sex	Sex (male/female)
3	9	cp	Chest pain type)
4	10	trestpbs	Resting blood pressure (mm Hg)
5	12	chol	Serum cholesterol (mg/dL)
6	16	fbs	Fasting blood sugar (120 mg/dL)
7	19	restecg	Resting electrocardiographic results
8	32	Thalach	Maximum heart rate achieved
9	38	Exang	Exercise-induced angina
10	40	Oldpeak	ST depression induced by exercise relative to rest
11	41	slope	The slope of the peak exercise ST segment
12	44	ca	Number of major vessels (0–3) colored by fluoroscopy
13	51	thal	3 = normal; 6 = fixed defect; 7 = reversible defect
14	58	num	Diagnosis of heart disease (angiographic disease status)

**Table 2:** Description of subparametric values for opted parameters.

Ordering by Scrutiny	Ordering by CD-Set	Parameters (Short Names)	Parameters (Full Names)	Values related to Parameters in CD-Set
1	3	age	Age in years	0–20, 21–40, 41–60, Above 60
3	9	cp	Chest pain type	1. Typical angina, 2. atypical angina, 3. non-anginal pain, 4. asymptomatic
4	10	trestpbs	Resting blood pressure (mm Hg)	90–200 mm Hg
5	12	chol	Serum cholesterol (mg/dL)	126–564 mg/dL
6	16	fbs	Fasting blood sugar (120 mg/dL)	120 mg/dL
8	32	Thalach	Maximum heart rate achieved	71–195
10	40	Oldpeak	ST depression induced by exercise relative to rest	0.0–5.6
11	41	slope	The slope of the peak exercise ST segment	1. upsloping, 2. flat, 3. downsloping
13	51	thal	3 = normal; 6 = fixed defect; 7 = reversible defect	1. normal, 2. fixed defect, 3. reversible defect

**Table 3:** Fuzzy-values-based ranges of opted parameters.

Selected Parameters	Relevant Values in CD-Set	Transformed Fuzzy Membership Grades
Age	0–20, 21–40, 41–60, 61–80	0–0.25, 0.2625–0.50, 0.5125–0.75, 0.7625–1.00
Chest pain type)	1, 2, 3, 4	0.25, 0.50, 0.75, 1.00
Resting blood pressure	90–200	0.45–1.00
Serum cholesterol	126–564	0.2234–1.0000
Fasting blood sugar	0, 120	0,1
Maximum heart rate achieved	71–195	0.3641–1.0000
Oldpeak	0.0–5.6	0–1
Slope	1, 2, 3	0.33, 0.66, 1.00
Thal	3, 6, 7	0.43, 0.86, 1.00

**Table 4:** Fuzzy membership corresponding to each ${\ddot{\partial }}^{ij}$.

${\ddot{\partial }}^{\mathrm{ij}}$	$\mu ({\ddot{\partial }}^{\mathrm{ij}})$	${\ddot{\partial }}^{\mathrm{ij}}$	$\mu ({\ddot{\partial }}^{\mathrm{ij}})$
${\ddot{\partial }}^{12}$	0.5	${\ddot{\partial }}^{13}$	0.7
${\ddot{\partial }}^{21}$	0.25	${\ddot{\partial }}^{22}$	0.50
${\ddot{\partial }}^{32}$	0.75	${\ddot{\partial }}^{42}$	0.57
${\ddot{\partial }}^{51}$	1.00	${\ddot{\partial }}^{61}$	0.42
${\ddot{\partial }}^{62}$	0.72	${\ddot{\partial }}^{72}$	0.66
${\ddot{\partial }}^{83}$	1.00	${\ddot{\partial }}^{92}$	0.86

**Table 5:** Fuzzy membership corresponding to each ${\ddot{\hbar }}^{i}$.

${\ddot{\hbar }}_{i}$	${\ddot{\hbar }}_{1}$	${\ddot{\hbar }}_{2}$	${\ddot{\hbar }}_{3}$	${\ddot{\hbar }}_{4}$	${\ddot{\hbar }}_{5}$	${\ddot{\hbar }}_{6}$	${\ddot{\hbar }}_{7}$	${\ddot{\hbar }}_{8}$
$\mu ({\ddot{\partial }}^{12})$	0.5	0.5	0.5	0.5				
$\mu ({\ddot{\partial }}^{13})$					0.7	0.7	0.7	0.7
$\mu ({\ddot{\partial }}^{21})$	0.25	0.25			0.25	0.25		
$\mu ({\ddot{\partial }}^{22})$			0.5	0.5			0.5	0.5
$\mu ({\ddot{\partial }}^{32})$	0.75	0.75	0.75	0.75	0.75	0.75	0.75	0.75
$\mu ({\ddot{\partial }}^{42})$	0.57	0.57	0.57	0.57	0.57	0.57	0.57	0.57
$\mu ({\ddot{\partial }}^{51})$	1.0	1.0	1.0	1.0	1.0	1.0	1.0	1.0
$\mu ({\ddot{\partial }}^{61})$	0.42		0.42		0.42		0.42	
$\mu ({\ddot{\partial }}^{62})$		0.72		0.72		0.72		0.72
$\mu ({\ddot{\partial }}^{72})$	0.66	0.66	0.66	0.66	0.66	0.66	0.66	0.66
$\mu ({\ddot{\partial }}^{83})$	1.0	1.0	1.0	1.0	1.0	1.0	1.0	1.0
$\mu ({\ddot{\partial }}^{92})$	0.86	0.86	0.86	0.86	0.86	0.86	0.86	0.86
$\mu ({\ddot{\hbar }}_{i})$	0.667	0.701	0.695	0.729	0.690	0.723	0.717	0.751

**Table 6:** Tabular Representation of Δ-set $S_{\Delta }$.

$\frac{{\ddot{\hbar }}_{i}}{\mu ({\ddot{\hbar }}_{i})}\setminus {\hat{p}}_{i}$	${\hat{p}}_{1}$	${\hat{p}}_{2}$	${\hat{p}}_{24}$	${\hat{p}}_{25}$	${\hat{p}}_{75}$	${\hat{p}}_{303}$
$\frac{{\ddot{\hbar }}_{1}}{0.667}$	0.2	0.3	0.0	0.4	0.6	0.7
$\frac{{\ddot{\hbar }}_{2}}{0.701}$	0.0	0.4	0.5	0.6	0.7	0.8
$\frac{{\ddot{\hbar }}_{3}}{0.695}$	0.3	0.5	0.3	0.0	0.4	0.5
$\frac{{\ddot{\hbar }}_{4}}{0.729}$	0.5	0.4	0.3	0.2	0.0	0.1
$\frac{{\ddot{\hbar }}_{5}}{0.690}$	0.0	0.2	0.3	0.4	0.5	0.6
$\frac{{\ddot{\hbar }}_{6}}{0.723}$	0.4	0.4	0.5	0.6	0.8	0.0
$\frac{{\ddot{\hbar }}_{7}}{0.717}$	0.3	0.6	0.4	0.4	0.5	0.2
$\frac{{\ddot{\hbar }}_{8}}{0.751}$	0.7	0.5	0.3	0.5	0.4	0.3

**Table 7:** Containment of ${\hat{p}}_{i}$ in approximate values of ${\ddot{\hbar }}_{i}$.

${\hat{p}}_{1}$	$\left(0.2,\frac{{\ddot{\hbar }}_{1}}{0.667}\right)$, $\left(0.3,\frac{{\ddot{\hbar }}_{3}}{0.695}\right)$, $\left(0.5,\frac{{\ddot{\hbar }}_{4}}{0.729}\right)$, $\left(0.4,\frac{{\ddot{\hbar }}_{6}}{0.723}\right)$, $\left(0.3,\frac{{\ddot{\hbar }}_{7}}{0.717}\right)$, $\left(0.7,\frac{{\ddot{\hbar }}_{8}}{0.751}\right)$
${\hat{p}}_{2}$	$\left(0.3,\frac{{\ddot{\hbar }}_{1}}{0.667}\right)$, $\left(0.4,\frac{{\ddot{\hbar }}_{2}}{0.701}\right)$, $\left(0.5,\frac{{\ddot{\hbar }}_{3}}{0.695}\right)$, $\left(0.4,\frac{{\ddot{\hbar }}_{4}}{0.729}\right)$, $\left(0.2,\frac{{\ddot{\hbar }}_{5}}{0.690}\right)$, $\left(0.4,\frac{{\ddot{\hbar }}_{6}}{0.723}\right)$, $\left(0.6,\frac{{\ddot{\hbar }}_{7}}{0.717}\right)$, $\left(0.5,\frac{{\ddot{\hbar }}_{8}}{0.751}\right)$
${\hat{p}}_{24}$	$\left(0.5,\frac{{\ddot{\hbar }}_{2}}{0.701}\right)$, $\left(0.3,\frac{{\ddot{\hbar }}_{3}}{0.695}\right)$, $\left(0.3,\frac{{\ddot{\hbar }}_{4}}{0.729}\right)$, $\left(0.3,\frac{{\ddot{\hbar }}_{5}}{0.690}\right)$, $\left(0.5,\frac{{\ddot{\hbar }}_{6}}{0.723}\right)$, $\left(0.4,\frac{{\ddot{\hbar }}_{7}}{0.717}\right)$, $\left(0.3,\frac{{\ddot{\hbar }}_{8}}{0.751}\right)$
${\hat{p}}_{25}$	$\left(0.4,\frac{{\ddot{\hbar }}_{1}}{0.667}\right)$, $\left(0.6,\frac{{\ddot{\hbar }}_{2}}{0.701}\right)$, $\left(0.2,\frac{{\ddot{\hbar }}_{4}}{0.729}\right)$, $\left(0.4,\frac{{\ddot{\hbar }}_{5}}{0.690}\right)$, $\left(0.6,\frac{{\ddot{\hbar }}_{6}}{0.723}\right)$, $\left(0.4,\frac{{\ddot{\hbar }}_{7}}{0.717}\right)$, $\left(0.5,\frac{{\ddot{\hbar }}_{8}}{0.751}\right)$
${\hat{p}}_{75}$	$\left(0.6,\frac{{\ddot{\hbar }}_{1}}{0.667}\right)$, $\left(0.7,\frac{{\ddot{\hbar }}_{2}}{0.701}\right)$, $\left(0.4,\frac{{\ddot{\hbar }}_{3}}{0.695}\right)$, $\left(0.5,\frac{{\ddot{\hbar }}_{5}}{0.690}\right)$, $\left(0.8,\frac{{\ddot{\hbar }}_{6}}{0.723}\right)$, $\left(0.5,\frac{{\ddot{\hbar }}_{7}}{0.717}\right)$, $\left(0.4,\frac{{\ddot{\hbar }}_{8}}{0.751}\right)$
${\hat{p}}_{303}$	$\left(0.7,\frac{{\ddot{\hbar }}_{1}}{0.667}\right)$, $\left(0.8,\frac{{\ddot{\hbar }}_{2}}{0.701}\right)$, $\left(0.5,\frac{{\ddot{\hbar }}_{3}}{0.695}\right)$, $\left(0.1,\frac{{\ddot{\hbar }}_{4}}{0.729}\right)$, $\left(0.6,\frac{{\ddot{\hbar }}_{5}}{0.690}\right)$, $\left(0.2,\frac{{\ddot{\hbar }}_{7}}{0.717}\right)$, $\left(0.3,\frac{{\ddot{\hbar }}_{8}}{0.751}\right)$

**Table 8:** Fuzzy membership $\zeta_{\Delta }^{D_{1}}$ for each ${\hat{p}}_{i}$.

${\hat{p}}_{i}$	$\zeta_{\Delta }^{D_{1}}\left({\hat{p}}_{i}\right)$
${\hat{p}}_{1}$	0.217050
${\hat{p}}_{2}$	0.294063
${\hat{p}}_{24}$	0.232288
${\hat{p}}_{25}$	0.275663
${\hat{p}}_{75}$	0.343900
${\hat{p}}_{303}$	0.278850

**Table 9:** Fuzzy membership $\zeta_{\Delta }^{D_{2}}$ for each ${\hat{p}}_{i}$.

${\hat{p}}_{i}$	$\zeta_{\Delta }^{D_{2}}\left({\hat{p}}_{i}\right)$
${\hat{p}}_{1}$	0.306081
${\hat{p}}_{2}$	0.414684
${\hat{p}}_{24}$	0.327569
${\hat{p}}_{25}$	0.388736
${\hat{p}}_{75}$	0.484964
${\hat{p}}_{303}$	0.393231

**Table 10:** Structural analysis of presented structure with pre-developed approaches.

Authors	Structures	Focus on Attributes	Focus on Subattributive Values	Data Set	Proper Criteria for Fuzzification of Fuzzy Parameters	Riesz Summability
Ça$\overset{˘}{g}$ man et al. [39]	fpfs-set	Yes	Ignored	Hypothetical	N/A	N/A
Yılmaz et al. [40]	fpfs-set	Yes	Ignored	Hypothetical	N/A	Yes
Kirişci [41,42]	fpfs-set	Yes	Ignored	CD-set	N/A	N/A
Riaz et al. [43]	fpfs-set	Yes	Ignored	Hypothetical	N/A	N/A
Zhu et al. [44]	fpfs-set	Yes	Ignored	Hypothetical	N/A	N/A
Rahman et al. [48]	fpfhs-set	Yes	Yes	Hypothetical	N/A	N/A
Proposed Study	fpfhs-set	Yes	Yes	CD-set	Adopted	Yes

**Table 11:** Structural analysis of presented structure with predeveloped approaches.

Authors	Structures	NOA	NOP	Ranking Based on Riesz Summability Method	Ranking Based on Other Adopted Method	Remarks
Kirişci [41]	fpfs-set	11	06	N/A	${\hat{p}}_{1}≻{\hat{p}}_{2}≻{\hat{p}}_{24}≻{\hat{p}}_{75}≻{\hat{p}}_{25}≻{\hat{p}}_{303}$	subattributive values are ignored.
Kirişci [42]	fpfs-set	11	06	N/A	${\hat{p}}_{75}≻{\hat{p}}_{24}≻{\hat{p}}_{25}≻{\hat{p}}_{1}≻{\hat{p}}_{2}≻{\hat{p}}_{303}$	subattributive values are ignored.
Proposed Study	fpfhs-set	09	06	${\hat{p}}_{75}≻{\hat{p}}_{2}≻{\hat{p}}_{303}≻{\hat{p}}_{25}≻{\hat{p}}_{24}≻{\hat{p}}_{1}$	${\hat{p}}_{75}≻{\hat{p}}_{2}≻{\hat{p}}_{303}≻{\hat{p}}_{25}≻{\hat{p}}_{24}≻{\hat{p}}_{1}$	Although values of both methods are different but they both proved analogous with similar ranking of patients.

## Data Availability

In this research, the data relating to attributes and their subattributes are taken from the Cleveland Data set (heart disease dataset), which is freely available online at (http://archive.ics.uci.edu/ml/datasets/Heart+Disease) (accessed on 3 October 2021).
